# Topical insulin in neurotrophic keratopathy after diabetic vitrectomy

**DOI:** 10.1038/s41598-024-60699-y

**Published:** 2024-05-14

**Authors:** Taher K. Eleiwa, Ahmed A. Khater, Abdelrahman M. Elhusseiny

**Affiliations:** 1 Department of Ophthalmology, Benha University, Benha, Egypt; 2 Department of Ophthalmology, Kasr. Al-Ainy Hospitals, Cairo University, Cairo, Egypt; 3 Department of Ophthalmology, University of Arkansas for Medical Sciences, Little Rock, AR USA

**Keywords:** Insulin, Neurotrophic, Corneal ulcer, Vitrectomy, Diabetes, Corneal diseases, Diabetes, Outcomes research, Drug safety

## Abstract

To assess the efficacy and safety of topical insulin (TI) for treating neurotrophic keratopathy (NK) within one-month post-diabetic vitrectomy (DV) compared to conventional non-invasive measures, we conducted this retrospective case-control study including all eyes that developed acute NK (stages 2 and 3) following DV between October 2020 and June 2023. The control group included NK cases managed with preservative-free lubricant eye drops and prophylactic topical antibiotics. In contrast, the study group included NK cases treated with TI [1 unit per drop] four times daily, in addition to the previously mentioned treatment. The primary outcome measure was time to epithelial healing. Secondary outcome measures included any adverse effect of TI or the need for amniotic membrane transplantation (AMT). During the study period, 19 patients with a mean age of 49.3 ± 8.6 years received TI versus 18 controls with a mean age of 52.5 ± 10.7 years. Corneal epithelial healing was significantly faster in the TI-treated group compared to controls, with a mean difference of 12.16 days (95% CI 6.1–18.3, P = 0.001). Survival analysis indicated that the insulin-treated group had 0% and 20% of NK stages 2 and 3, respectively, that failed to achieve corneal epithelial healing, compared to 20% and 66.7% for the control group (P < 0.001). In the control group, two eyes required AMT due to progressive thinning. Additionally, three patients in the control group, progressing to stage 3 NK, were switched to TI, achieving healing after a mean of 14 days. No adverse effects were reported in the TI-treated group. Our study suggests that TI can effectively and safely promote the healing of NK after DV.

## Introduction

Corneal nerves play a vital role in maintaining the homeostasis of the ocular surface^1^. Not only mediating sensory reflexes such as blinking and lacrimation but also corneal nerves critically preserve the integrity of corneal epithelium and the nerves themselves via producing trophic factors^1–3^. An insult anywhere from the trigeminal nerve nucleus to the terminal nerve endings of the nasociliary nerve can disrupt this homeostasis and lead to corneal hypoesthesia and neurotrophic keratopathy (NK)^1,4^. The pathogenesis of NK has been associated with infectious, inflammatory, toxic, and iatrogenic etiologies such as ocular herpetic infection, ocular or neurologic surgery, trauma, chemical burns, diabetes, and dry eye disease^5,6^.

In diabetic keratopathy, several corneal changes have been reported, including abnormal basement membrane structure, poor epithelial adherence, hypothesia, and alterations in the corneal stroma, Descemet membrane, and corneal endothelium^7^. Also, NK has been reported as a rare complication of pan-retinal photocoagulation (PRP) and trans-scleral cyclophotocoagulation^8–12^. The primary suggested mechanism entails thermal injury to the long ciliary nerve branches as they enter the suprachoroidal space at the positions corresponding to 3 and 9 o'clock on the retina^10,13^. In diabetic patients, NK may present as a persistent epithelial defect refractory to conventional measures, predisposing to microbial keratitis and/or stromal melting/scarring with subsequent perforation/blindness^14,15^. Thus, rapid corneal re-epithelialization is needed to restore the corneal surface integrity.

Various treatment options have been proposed in managing NK, such as preservative-free lubrication (PF-L), withdrawal of epitheliotoxic medication, prophylactic antibiotics, applying of bandage contact lenses, using hemoderivatives, topical insulin (TI), recombinant nerve growth factor (rNGF) or epidermal growth factor, or amniotic membrane transplant (AMT), or corneal neurotization^6,16–19^. Topical insulin (TI) has been reported to effectively promote the healing of persistent corneal epithelial defects, including NK^20–29^.

Our study explored the effectiveness and safety of TI as a first-line treatment in managing naïve acute-onset NK after diabetic vitrectomy.

## Methods

### Study design and participants

We conducted a retrospective chart review of adult diabetic patients (> 18 years of age) diagnosed with stages 2 or 3 NK within one month after diabetic vitrectomy. The study compared those who received TI as a first-line treatment versus controls with matched baseline characteristics who underwent the conventional non-invasive measures. The study was conducted at the Department of Ophthalmology in Benha University Hospital from October 2020 to July 2023.

Neurotrophic keratopathy was defined as stage 2 (persistent epithelial defect) or stage 3 (corneal ulcer) according to published criteria^30^. Informed consents for participation in the study were obtained from all patients. This study complied with the tenets of the Declaration of Helsinki and was approved by the Ethics Committee of Benha University (RC34-5-2023).

### Inclusion and exclusion criteria

We included patients with an established diagnosis of NK (ICD10: H16.2) in stage 2 or 3. NK was graded based on slit-lamp and fluorescein stain findings—stage 2: epithelial defect (with or without a rim of loose epithelium) without stromal ulceration, and stage 3: corneal ulceration and/or stromal lysis.

The corneal sensitivity test was evaluated using a sterile cotton thread in the center and four quadrants of both corneas. The presence of corneal sensitivity was determined when the patient blinked or described a touch sensation. Conversely, the absence of sensitivity was noted when the patient reported no sensation, or no reaction was observed.

We excluded patients with a history of herpetic eye disease, active ocular infection or inflammation unrelated to NK, chemical burn, limbal stem cell deficiency, lagophthalmos, ocular surgeries other than recent pars-plana vitrectomy (PPV), or combined phaco-PPV, or other ocular diseases such as glaucoma or severe vision loss in the affected eye(s). Additionally, we excluded patients with chronic use of preservative-containing eye drops.

### Data collection

We collected data from patients’ medical records and slit-lamp photographs (with and without fluorescein staining). Each patient received a detailed explanation regarding potential alternatives, risks, and benefits associated with the off-label use of insulin.

### Treatment protocol

Per hospital protocol, post-vitrectomy medications include moxifloxacin 0.5% (Vigamox, Alcon Inc., Fort Worth, TX) and prednisolone acetate 1% (Pred Forte; Allergan, Irvine, CA). After the diagnosis of NK, both eye drops were gradually tapered. Additionally, PF-L (Sodium hyaluronate 1 mg + Carboxymethyl cellulose 5 mg + Glycerin 9 mg; polyfresh extra single dose unit; Orchidia Pharmaceutical Ind., Cairo, Egypt) was added six times daily.

TI drops were compounded at the hospital pharmacy by diluting 2.5 ml of insulin (Actarapid; Novo Nordisk A/S, Søborg, Denmark) containing 250 units of insulin with 2.5 ml of artificial tears containing a propylene glycol base in a commercially available applicator bottle. This resulted in a concentration of 50 units per milliliter (U/ml) of insulin. Given the standard volume of commercial eye drop bottles (5 ml), each drop dispensed approximately 20 µl, thus delivering roughly 1 unit of insulin per drop from the 50U/ml solution. These drops were preserved at a low temperature (2 °C). TI was self-administered by patients at a dosage of 1 eye drop four times daily.

### Study endpoints

The study's endpoints included complete corneal healing, monitoring for adverse events, and assessing the need for AMT owing to progressive corneal thinning or stromal melting despite good compliance. Complete corneal healing was defined as the absence of fluorescein staining in the ulcer area.

### Statistical analysis

We conducted the statistical analysis using the Statistical Package for the Social Sciences version 25 (SPSS Inc.; Chicago, IL). For descriptive purposes of the study sample, we presented the mean and standard deviation (SD) for continuous variables. The data distribution was analyzed using the Shapiro–Wilk test. We calculated the Cohen's d to estimate the effect size.

We compared the outcome measure "time till healing" between groups using the independent samples t-test and Kaplan–Meier analysis. Additionally, we conducted a factorial analysis to explore whether the "time till healing" outcome variable depended, in part, on the stage of NK in both groups. The resulting significance level was set at P < 0.05.

## Results

During the study period, 37 eyes (37 patients) out of 737 diabetic vitrectomy surgeries were included, resulting in a 3-year NK (stages 2 and 3) prevalence rate of 5.02%. The data exhibited a normal distribution without significant outliers. The Cohen's d was greater than 0.8, indicating a large effect size.

The TI-treated group included 19 eyes of 19 patients (11 males and 8 females) with a mean age of 49.3 ± 8.6 years (range: 39–70 years). The control group included 18 eyes of 18 patients (10 males and 8 females) with a mean age of 52.5 ± 10.7 years (range 28–72). No significant differences were observed in age, gender, type of diabetes mellitus, hemoglobin A1c levels, or NK stage distribution at the time of diagnosis between the two groups (P > 0.05, see Table 1). Preoperative corneal sensitivity results indicated a diminished blink response in 13 eyes (59%) of the TI-treated group and 14 (60.1%) in the control group.

**Table 1:** Demographics and clinical pre-operative characteristics of the study groups.

	Control group (n. 18 eyes)	Study group (n. 19 eyes)	P value
Age	52.5 ± 10.7	49.3 ± 8.6	0.30
Gender			
Male	10	11	0.57
Female	8	8	
DM type			
Type 1	2	2	0.67
Type 2	16	17	
HBA1c	8.5 ± 1.6	8.4 ± 1.05	0.82
Indication of vitrectomy			
Advanced diabetic retinopathy	7	8	0.52
Tractional retinal detachment	5	4	
Vitreous hemorrhage	6	7	
Silicone tamponade	14	13	0.19
Endolaser panretinal photocoagulation	100%	100%	1.0
Time till NK diagnosis (days)	10.6 ± 4.3	11.9 ± 4.5	0.63
NK stage			
2	15	14	0.98
3	3	5	

*DM* Diabetes Mellitus, *HBA1c* Hemoglobin A1C, *NK* Neurotrophic keratopathy.

Corneal epithelial healing occurred significantly faster in the TI-treated group compared to the control group, with a mean difference of 12.16 days (95% CI 6.1–18.3, P = 0.001, see Table 2). Additionally, the mean number of days until corneal healing was significantly lower in stage 2 compared to stage 3, with a mean difference of 13.3 days (95% CI 6.5–20.1, P = 0.001) and 29.4 days (95% CI 24.2–34.6, P < 0.0001, see Table 2), respectively. Factorial analysis revealed that the stage of NK affected 40% of the healing duration in both groups (P = 0.011).

**Table 2:** Outcomes of neurotrophic keratopathy (NK) treatment in both groups.

	Control group	Study group	
Time till NK healing (days)	23.1 ± 11.6	10.9 ± 6.8	**0.001**
Time till NK healing (days)			
Stage 2	21.7.0 ± 11.1	8.4 ± 3.2	**0.001**
Stage 3	48.0 ± 3.6	18.6 ± 2.5	** < 0.0001**
Failure of healing	5	1	–
Need of AMT	2	0	–
Need of Bandage contact lens	5	1	–

Significant values are in bold.

*AMT* amniotic membrane transplantation.

**Figure 1:**
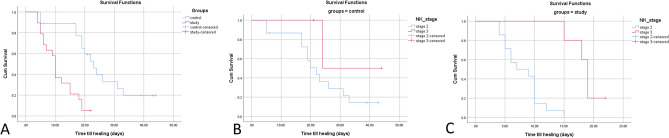
(**A**) Kaplan–Meier curves for days till epithelization in patients with neurotrophic keratopathy (NK) in both groups. The cumulative (Cum) survival ratio was 94.7% in the insulin-treated group versus 72.2% in the control group (Log Rank test P < 0.0001). Five cases were censored in the control group versus 1 case in the study group. (**B**, **C**) curves showed that the control group showed 80% and 33.3% of NK stages 2, and 3, respectively achieved corneal epithelial healing versus 100% and 80% for the insulin-treated group (Log Rank test, P < 0.001).

The Kaplan–Meier survival analysis demonstrated that 1 case (5.3%) in the TI-treated group did not achieve corneal healing, compared to 5 cases (27.8%) in the control group (Log Rank test, P < 0.0001, see Fig. 1A). In terms of survival analysis factored by NK stage, the control group exhibited that 20% and 66.7% of NK stage 2 and 3, respectively, failed to achieve corneal epithelial healing, in contrast to 0% and 20% for the TI-treated group (Log Rank test, P < 0.001, see Fig. 1B,C). Notably, compared to the preoperative data, corneal sensation was qualitatively restored in the healed ulcer areas in the TI-treated group, while no difference was observed in the controls.

**Figure 2:**
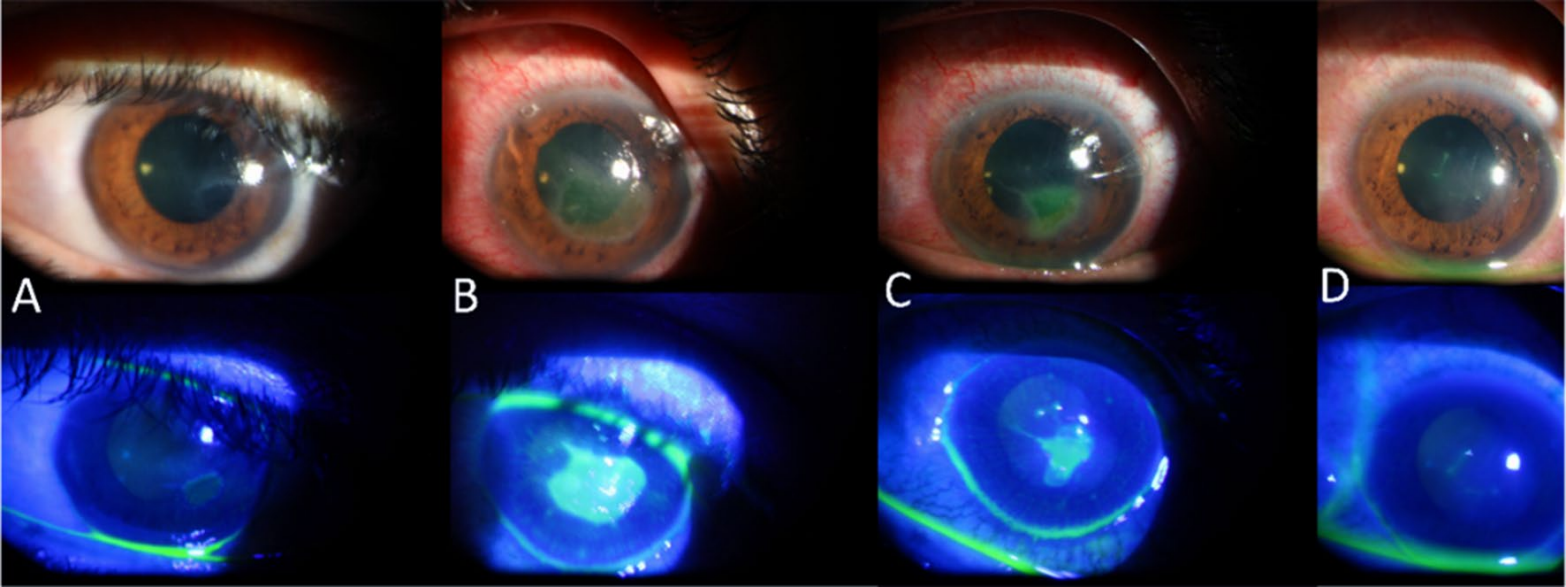
Slit-lamp photography of the left cornea in a 39-year-old male who had a vitrectomy performed for diabetic tractional retinal detachment. The patient was referred to the cornea clinic and diagnosed with stage 2 neurotrophic keratopathy (NK) by the 9th postoperative day (**A**). His treatment was modified, and conventional treatment was started. Initial stabilization was observed, but NK had progressed to stage 3 (**B**) by the 6th day of treatment. Topical insulin was added, and a bandage contact lens was applied. The ulcer was reduced in size after five days of treatment (**C**), and complete healing was observed after a further 14 days (**D**).

At the discretion of the cornea specialist, corneal epithelial debridement and tectonic AMT were performed in 2 cases (stage 3 NK) in the control group due to progressive corneal thinning (stromal melting). For the other 3 cases in the control group that progressed from stage 2 to stage 3 NK (corneal ulcer without stromal melting), TI and bandage contact lenses were added, and healing was achieved after a mean of 14 ± 7.1 days of treatment (see Fig. 2). A bandage contact lens was applied in the TI-treated case with a persistent residual epithelial defect, and healing was completed after a further five days of treatment.

Corneal clarity was maintained in all cases in the TI-treated group compared to 83% of cases in the control group. Superficial corneal vascularization and faint corneal opacity were observed in two controls who underwent the AMT.

No adverse events were reported for the use of TI drops.

## Discussion

Maintaining corneal clarity in diabetic patients following diabetic vitrectomy is crucial for improving visual outcomes and facilitating the diagnostic and therapeutic visualization of the retina. This study underscores the vital role of corneal sensitivity testing after diabetic vitrectomy for the early diagnosis of NK. Moreover, the findings indicate that the early recognition of stages 2 and 3 NK, coupled with the initiation of TI, promoted corneal healing without significant residual scarring. This aligns with a recent retrospective study reporting faster healing rates in stage 2 NK compared to stage 3 using TI^26^.

Regrading corneal epithelial healing, several studies have reported the therapeutic role of TI in corneal re-epithelialization. Diaz-Valle and colleagues reported a mean time of 34.8 ± 9.9 days (median 23; range 7–114) for re-epithelialization using TI (1 IU/mL 4 times daily) in 21 patients with refractory persistent epithelial defects^25^. This wide range in their study could be attributed to the heterogeneity of their study group.

Bastion and Ling^31^, in a retrospective study, reported faster corneal re-epithelialization within 48 h using TI (1 IU/mL 4 times daily) in 15 eyes of 14 patients who underwent corneal epithelial debridement during pars-plana vitrectomy compared to conventional treatment (72 h). Similarly, two studies reported 100% corneal re-epithelization within 36–72 h using TI (0.5 IU/mL 4 times daily) in the eyes of patients with iatrogenic corneal epithelial defect after PPV.

In patients with recurrent epithelial erosions, Esmail and colleagues^32^ reported that TI can promote corneal re-epithelization and decrease recurrences. In the context of addressing dry eye disease, TI displayed comparable therapeutic outcomes to artificial tears, although it appeared to potentially increase tear break-up time^33,34^. Furthermore, TI demonstrated efficacy in various ocular conditions, including NK, persistent epithelial defects, and exposure keratopathy, leading to swift re-epithelialization when traditional treatment regimens were ineffective^35^. These findings underscore the potential of TI as a valuable therapeutic modality for a range of ocular conditions.

The reported prevalence and timing of iatrogenic NK are inconsistent in the literature. Upon reviewing the existing studies, NK after retinal surgeries was found to develop between four weeks to 10 months postoperatively^8–11,36–39^. A recent retrospective study reported a 9-year prevalence rate of 2.9% following rhegmatogenous retinal detachment surgery^8^. However, it is crucial to note that they excluded diabetes from their cohort. Other reports indicated a prevalence ranging from 0.01 to 0.05% following other etiologies, such as herpetic keratitis and postoperative procedures for trigeminal neuralgia^40,41^.

In the current study, we found a 3-year prevalence of 5% for acute-onset NK after diabetic vitrectomy. It is noteworthy that we also excluded patients who required intraoperative epithelial debridement. The higher prevalence reported in this study may be attributed to the routine corneal sensitivity testing during follow-ups after diabetic vitrectomy. Without such surveillance, the early stages of NK might be misinterpreted as dry eye disease, potentially leading to delayed diagnosis and treatment. Nevertheless, possible confounding factors in our study, such as variability of vitrectomy approach, duration of surgery, NK severity in each stage, treatment compliance, the decision-making process for implementing adjunctive treatments, and duration of follow-up, may introduce bias in the estimated prevalence and the study outcomes. To address these potential confounders and enhance the robustness of the findings, future studies should aim to account for these factors and consider conducting meta-analyses for further validation.

Further, in vivo studies are warranted to gain a deeper understanding of the mechanisms and timing of NK after diabetic vitrectomy.

Insulin and insulin-like growth factor-1 (IGF-1) are present in human tears and likely play a vital role in maintaining ocular surface homeostasis^42^. Although the corneal epithelium has been shown to be an insulin-insensitive tissue and does not require insulin for glucose uptake, recent studies reveal that insulin, when applied topically, promotes corneal wound healing in diabetic keratopathy^42^. Additionally, insulin is implicated in restoring circadian rhythm, diurnal control of proliferation, and regulating mitochondrial homeostasis in corneal epithelial cells^43^. The underlying mechanism involves the homodimeric insulin receptor (INSR), supported by the insulin mimic monoclonal antibodies-20 (MA-20), which specifically binds to homodimeric INSR but not insulin hybrid receptor (Hybrid–R). Insulin treatment, acting through homodimeric INSR, plays a crucial role in regulating key cellular functions necessary for corneal cell growth and survival in diabetes^44^.

Addressing the neurotrophic pathology is crucial for promoting and stabilizing the corneal healing and preventing potential recurrences^45,46^. The presumed hypothesis of NK after retinal surgeries involves the presumed injury to the long ciliary nerves at 3 and 9 o’clock positions^10,13^. In addition to the reduced corneal nerve density, the reduced IGF-mediated corneal epithelial cell proliferation plays a vital role in the pathogenesis of NK in diabetic corneas.

Another critical factor hindering ocular surface healing in diabetic patients is the hyperosmolar tear film, resulting from impaired lacrimal gland secretion and the presence of advanced glycation end products from chronic hyperglycemia. Topical insulin has been reported to improve dry eye symptoms in diabetic patients, inducing IGF-1-mediated corneal nerve regeneration and corneal epithelial proliferation.

The multifactorial relationship between diabetic keratopathy and TI, as aforementioned regarding the functions of insulin on the ocular surface, may explain why TI, in this study, was superior to PF-L in healing NK. This could also account for the relative increase in the corneal sensitivity observed in the TI-treated group compared to the control group. Further, In-vivo confocal microscopic studies are warranted to understand the chronological histopathological changes in NK before and after treatment.

The patient from the TI-treated group who had a residual small corneal epithelial defect at four weeks and achieved complete healing after five days on bandage contact lens highlights the importance of careful attention to diabetic patients with denuded epithelia, as their healing process can be hindered despite treatment. In five cases, PF-L alone was inadequate to close the epithelial defects.

Figure 2 illustrates the initial progression in one case in the control group that was switched to TI after six days of treatment with PF-L. This case further emphasizes the role of insulin in addressing the NK pathology in diabetic individuals. Importantly, no adverse events were reported for TI, such as microbial keratitis and hypoglycemic episodes. Similarly, Bastion et al. and Bartlett et al., reported no adverse event or clinical toxicity associated with TI in their studies^31,47,48^.

Our study has some limitations that should be considered when interpreting the results. Firstly, a small sample size necessitates cautious interpretation, and future studies with larger cohorts are essential to validate the findings. Nevertheless, the calculated large effect size of the current sample size made the statistical utility of the t-test feasible. Secondly, qualitative corneal sensitivity testing is less informative, and future studies should incorporate quantitative corneal sensitivity testing, such as the Cochet-Bonnet esthesiometer. Thirdly, we did not compare TI with other potentially effective therapies for NK (e.g., autologous serum, plasma rich in growth factors eye drops (PRGF), and topicalrNGF drops). However, TI offers advantages in terms of rapid dispensation to the patient, widespread availability, low cost, excellent tolerance, and no reported adverse ocular effects^25^. Moreover, we did not compare different concentrations of insulin, which may influence results, as suggested in a different study^21^. In addition, the retrospective short-term nature of our study did not allow us to determine the potential application of TI as a prophylactic measure against the development of NK after diabetic vitrectomy and NK recurrences. Finally, no conclusions could be made from our study regarding the interaction between NK treatment and postoperative retinal status on visual outcomes. Larger prospective studies are needed to better understand the visual outcomes in eyes with NK after diabetic vitrectomy based on retinal characteristics and NK severity.

## Conclusions

While recognizing the rationale for surgical management, such as AMT, in NK, the decision to apply it remains subjective. Our clinical observation suggests that TI is notably more effective than conventional approaches. Since incorporating TI into our protocols, we have observed a reduced need for surgical interventions in cases of NK following diabetic vitrectomy at our center. As a result, we have modified our treatment algorithm for NK stages 2 and 3, designating TI as the first line of treatment. Although this study serves as a pilot to support our clinical impression of TI's efficacy, randomized prospective studies with larger sample sizes and accounting for the aforementioned confounding factors are imperative to validate these initial findings. These studies will provide a more substantial evidence base regarding the efficacy of varying concentrations of TI compared to autologous serum, PRGF, and rNGF in treating NK after diabetic vitrectomy.

## Data Availability

All data generated or analyzed during this study are available upon request from the corresponding author.
